# Clinical Decision-Making Case: Febrile Infant

**DOI:** 10.5070/M5.52290

**Published:** 2026-01-31

**Authors:** Carrie Maupin, Ambika Anand, Grace Hickam, Danielle Nesbit

**Affiliations:** * Virginia Commonwealth University, Department of Emergency Medicine, Richmond, VA

## Abstract

**Audience:**

This clinical decision-making (CDM) case is intended for emergency medicine residents of all levels, medical students, and fellows preparing for standardized oral board exams.

**Introduction:**

Fever in a neonate (infant <28 days old) is a medical emergency due to the high risk of serious bacterial infections (SBIs) like meningitis, sepsis, or urinary tract infections (UTIs).^1^–^3^ Compared with older infants and children, neonates have immature immune responses, reduced ability to localize infection, and limited physiologic reserve, which contribute to rapid clinical deterioration and increased morbidity and mortality when invasive infection is present.^1^,^3^

Importantly, clinical presentation in this age group is often subtle and nonspecific. Neonates with life-threatening infections may appear well or only mildly ill on initial examination, with symptoms such as poor feeding, irritability, or decreased urine output serving as early but easily overlooked warning signs.^1^,^4^ As a result, reliance on appearance or focal examination findings alone is insufficient to safely exclude SBI in febrile neonates.

Current evidence supports a standardized approach to the evaluation of neonatal fever. This includes a complete sepsis workup—consisting of blood, urine, and cerebrospinal fluid studies—along with early administration of empiric, age-appropriate intravenous antibiotics and hospital admission for close monitoring.^1^–^3^

This clinical decision-making case is designed to reinforce these foundational principles within the context of an emergency department presentation. It emphasizes early recognition of neonatal fever as a high-risk condition, systematic diagnostic reasoning, timely initiation of empiric therapy, and appropriate disposition to a higher level of care. Learners are challenged to clearly articulate their clinical reasoning and management decisions in a high-stakes environment that mirrors real-world emergency medicine practice.

**Educational Objectives:**

By the end of this CDM case, learners will be able to: 1) demonstrate familiarity with the CDM case format, 2) recognize the critical importance of fever in a neonate and initiate a thorough evaluation, 3) develop an appropriate differential diagnosis and understand the workup for febrile neonates, 4) identify and justify the appropriate diagnostic studies and interpret their findings in the context of a neonate with fever, 5) justify a treatment plan and understand the critical disposition of a neonate with fever.

**Educational Methods:**

The case will be presented as a CDM case with questions posed by the examiner. Learners will be asked to list the history, physical exam findings, differential diagnosis, diagnostic studies, treatments, and final diagnosis in response to the examiner’s prompts.

**Research Methods:**

Learners’ performance will be evaluated using standardized oral board scoring guidelines. Efficacy will be assessed through feedback from both learners and faculty, focusing on knowledge acquisition and application in a high-stakes environment. Pre- and post-case surveys or performance scoring may be used for evaluation.

**Results:**

Preliminary assessments from learners demonstrated improved confidence in managing febrile neonates after completing the case, with a focus on early recognition and appropriate escalation of care.

**Discussion:**

Neonatal fever is a high-risk scenario requiring prompt, appropriate management. This case reinforced the importance of early sepsis recognition, comprehensive evaluation, and timely treatment. Learners benefited from exposure to the CDM Case format aiding in their exam preparation.

**Topics:**

Neonatal fever, sepsis, meningitis, pediatric emergency management, antibiotic management, ABEM Certifying Exam, clinical decision-making case.

## USER GUIDE

**Table:** 

List of Resources:	
Abstract	1
User Guide	3
For Examiner Only	5
Certifying Exam Assessment	12
Stimulus	14
Debriefing and Evaluation Pearls	22

**Learner Audience:**

This case is directed toward medical students, interns, and junior and senior residents.

**Time Required for Implementation:**

Case: CDM cases are 10 min per American Board of Emergency Medicine (ABEM) standard.

Debriefing: 5–10 minutes

**Recommended number of learners per instructor:**

1–2

**Topics:**

Neonatal fever, sepsis, meningitis, pediatric emergency management, antibiotic management, ABEM Certifying Exam, clinical decision-making case.

**Objectives:**

By the end of this CDM case, learners will be able to:

1. Demonstrate familiarity with the CDM case format.
2. Recognize the critical importance of fever in a neonate and initiate a thorough evaluation.
3. Develop an appropriate differential diagnosis and understand the workup for febrile neonates.
4. Identify and justify the appropriate diagnostic studies and interpret their findings in the context of a neonate with fever.
5. Justify a treatment plan and understand the critical disposition of a neonate with fever.

### Linked objectives, methods and results

Objectives are achieved using a CDM case format in order to mimic the high-stakes environment of an oral board exam, specifically the CDM case. This format encourages learners to think critically and articulate their decision-making process, preparing them for actual clinical scenarios. Learners are expected to recognize the febrile neonate based on the abnormal vital signs and begin a septic work-up, as per the American Academy of Pediatrics (AAP) guidelines, including performing a lumbar puncture to rule out meningitis.^2^ Leaners are also expected to develop a differential diagnosis that covers the most common diagnoses in this patient population, such as meningitis, pneumonia, or UTIs.^1^ Throughout the case, learners are asked to justify the diagnostic studies ordered, historical questions asked, and physical exam performed. Finally, learners are asked to choose an appropriate final diagnosis and disposition based on the patient information gathered throughout the case. The conceptual framework revolves around active learning through case-based simulation, enhancing diagnostic reasoning and clinical decision-making skills.

### Recommended pre-reading for instructor

Any resource reviewing neonatal fever and/or meningitis, such as:

- Rodriguez DM, Nesiama JO, Wang VJ. Fever and serious bacterial illness in infants and children. In: Tintinalli JE, Ma O, Yealy DM, Meckler GD, Stapczynski J, Cline DM, Thomas SH. eds. *Tintinalli’s Emergency Medicine: A Comprehensive Study Guide*. 9^th^ ed. McGraw- Hill Education; 2020.
- Leazer RC. Evaluation and management of young febrile infants: An overview of the new AAP Guideline. *Pediatr Rev*. 2023;44(3):127–138. https://doi.org/10.1542/pir.2022-005624
- Mahajan P, Smitherman HF, Macias CG. The febrile neonate (28 days of age or younger): Initial management. In: UpToDate, Connor RF (Ed.), Wolters Kluwer. 2024. https://www.uptodate.com/contents/the-febrileneonate-28-days-of-age-or-younger-initialmanagement?search=neonatal%20fever&source=search_result&selectedTitle=3%7E150&usage_type=default&display_rank=3#H877598001
- Long B. Pediatric meningitis: Pearls and pitfalls. In: emDocs, Koyfman A, Alerhand, S, eds. 2015. https://www.emdocs.net/pediatric-meningitis-pearls-and-pitfalls/

### Results and tips for successful implementation

This CDM case was implemented during the annual clinical skills examination for our PGY-3 emergency medicine residents in April 2025. It was conducted in a one-on-one format, with each resident individually evaluated by a faculty member. While the case was used as part of a formal testing day, it is well-suited for any one-on-one evaluation setting and does not require a designated exam day for implementation. A total of 10 residents participated in the session.

Learners were assessed using a rubric specifically developed for this case, with performance categorized into five levels: Outstanding (23–25), Proficient (19–22), Adequate (15–18), Needs Improvement (10–14), and Inadequate (0–9). All residents passed the exercise, with scores ranging from 17 to 25. This allowed faculty to gauge clinical reasoning and communication in a standardized yet flexible way. Feedback was provided to each resident by the faculty member after the case was completed.

Feedback from learners led to minor but meaningful modifications in the case design. Specifically, “bacteremia” was added as a potential diagnosis on the differential, and “Cefotaxime” was included as an antibiotic option. These changes improved clinical relevance and aligned better with resident thought processes. Overall, residents found the case to be a fair and accurate representation of a CDM case, and they felt the level of difficulty was appropriate for their training level. For programs implementing this case, ensuring a one-on-one format and being open to iterative feedback are key strategies to enhance educational impact.

## Figures and Tables

**Table:** 

**I. History**		**Yes**	**No**
**1a**	Maternal infections in pregnancy		
**1b**	Urinary output		
**2a**	Provides appropriate rationale		
**2b**	Provides appropriate rationale		
**II. Physical Examination**			
**3a**	Examination of the fontanelle		
**3b**	Lung auscultation		
**3c**	Abdominal exam		
**4**	To assess for signs of dehydration or increased intracranial pressure.		
**III. Differential Diagnosis**			
**5a**	Diagnosis 1 - Meningitis, urinary tract infection, pneumonia, viral illness, bacteremia		
**5b**	Diagnosis 2 - Meningitis, urinary tract infection, pneumonia, viral illness, bacteremia		
**5c**	Diagnosis 2 - Meningitis, urinary tract infection, pneumonia, viral illness, bacteremia		
**IV. Diagnostic Studies**			
**6a**	Lumbar puncture What CSF studies would you order and why?		
**6b**	CXR		
**6c**	Urinalysis		
**7a**	Provides appropriate rationale		
**7b**	Provides appropriate rationale		
**V. Treatment and Other Actions**			
**8a**	Antibiotics (ampicillin + Gentamicin OR Cefepime OR Cefotaxime)		
**8b**	Tylenol		
**9**	Provides appropriate rationale		
**10**	IV ampicillin + gentamicin OR cefepime OR cefotaxime		
**VI. Broad Principles**			
**11**	Coverage for HSV (with acyclovir) should be considered when there is a maternal history of genital HSV lesions or fevers surrounding delivery and in infants who present with vesicles, seizures, or hypothermia.		
**VII. Final Diagnosis**			
**12**	Meningitis		
**VIII. Disposition**			
**13**	Admission to the pediatric intensive care unit (PICU or Neonatal ICU		
**14**	Rationale for admission to ICU level care		
**IX. Transitions of Care**			
**15a**	Provide sign out to the admitting team/explain diagnosis to the patient’s family (at least brief history and physical exam, diagnostic findings, diagnosis, and treatments given).		
**15b**	Discuss diagnosis and management plan with patient’s parent		

**Table:** 

**#1**	**Emergency Department Admitting Form**
**#2**	**Historical Information**
**#3**	**Physical Exam Findings**
**#4**	**Diagnostic Studies**
**#5**	**Chest X-ray**

**Table:** Clinical Decision-Making Candidate Task Sheet

**CASE PARAMETERS**
This is a 15-minute case You will interact with two examiners. This is an interview style without role playing; you should simply reply to the questions asked. You may be interrupted to move you through the case; this is not a reflection of your performance.
**PATIENT INFORMATION**
2-week-old male arriving by private vehicle with his mother with a chief complaint of fever. Patient’s mother reports that the infant has been more fussy than usual, with decreased feeding. No vomiting or diarrhea noted. No known sick contacts.
**VITAL SIGNS**
BP:70/40 mmHg, P: 190 bpm, R:50 rpm, T: 38.6°C, 02Sat: 98% on room air
**TASK STATEMENT**
Your tasks are as follows: List pertinent elements of a focused history and physical exam Develop an appropriate differential and/or provisional diagnosis Select and interpret appropriate studies Articulate appropriate patient management including discharge instructions

**STIMULUS 1:** Emergency Department Admitting Form

Patient Information	
**Patient Name**	***
**Age**	2-weeks-old
**Gender**	Male
**Method of Arrival**	Private vehicle
**General Appearance/History of Present Illness**	Mother reports that the baby has been more fussy than usual, with decreased feeding. No vomiting or diarrhea noted. No known sick contacts.
**Vital Signs on ED Arrival**	BP: 70/40 P: 190 R: 50 T: 38.6°C O2 sat: 98% on room air Weight: 4kg

**STIMULUS 2:** Historical Information

**History of Present Illness/Description of Event**	
Patient’s mother noted the fever today, measured at 100.6° F at home (axillary). He has been eating slightly less than usual and has had 2 wet diapers today. Mother denies any known sick contacts	
**History**	
**Past Medical History**	Born at 38 weeks 4 days and born by normal spontaneous vaginal delivery Mother did not receive any prenatal care and was found to be positive for Group B streptococcus (GBS) at the time of birth. She was given antibiotics at the time of birth. He received his recommended shots at birth
**Past Surgical History**	None
**Medications**	None
**Allergies**	No Known Drug Allergies

**STIMULUS 3:** Physical Exam Findings

Physical Examination	
**General**	The patient is an irritable but consolable infant
**HEENT**	Fontanelle is soft and flat, tympanic membranes are clear, no discharge from the eyes Mucous membranes are dry
**Respiratory**	Lungs are clear to auscultation in all fields
**Cardiac**	Cardiac exam shows tachycardia with a regular rhythm
**Abdominal**	Abdomen is soft and non-distended; there is no hepatosplenomegaly
**Extremities**	There is no cyanosis or edema in the extremities
**Neurologic**	Patient is moving all extremities with good tone throughout
**Dermatologic**	There are no rashes or jaundice

**STIMULUS 4 t8-jetem-11-1-ce1:** Diagnostic Studies: Pertinent Positives/Negatives

**CBC**	WBC	18 × 10^3^/mm^3^
	Hgb	14.2 g/dL
	Hct	46%
	Platelets	340/mm^3^
	Differential	
	Neutrophils	70%
	Lymphocytes	20%
	Monocytes	5%
	Eosinophils	3%
	Bands	2%

**BMP**	Na	143 mEq/L
	K	4.2 mEq/L
	Cl.	101 mEq/L
	CO_2_	22 mEq/L
	BUN.	7 mg/dL
	Cr.	0.3 mg/dL
	Glucose	90 mg/dL

**Urinalysis**	Appearance	Cloudy
	Color	Yellow
	Glucose	Negative
	Ketones	Negative
	Sp Gravity	1.008
	Blood	Negative
	pH	6.3
	Protein	Negative
	Nitrite	Negative
	Leukocyte	Negative
	WBC	<5
	RBC	<2
	Squamous Cells	Rare
	Bacteria	None seen

**LP Results**	Color	Clear
	WBCs	200 cells/mm^3^ (neutrophilic Predominance)
	RBCs	10 cells/mm^3^
	Glucose	30 mg/dL
	Protein	110 mg/dL
	Gram Stain	Positive for gram positive cocci
	Cerebral spinal fluid culture pending	

**STIMULUS 5:**
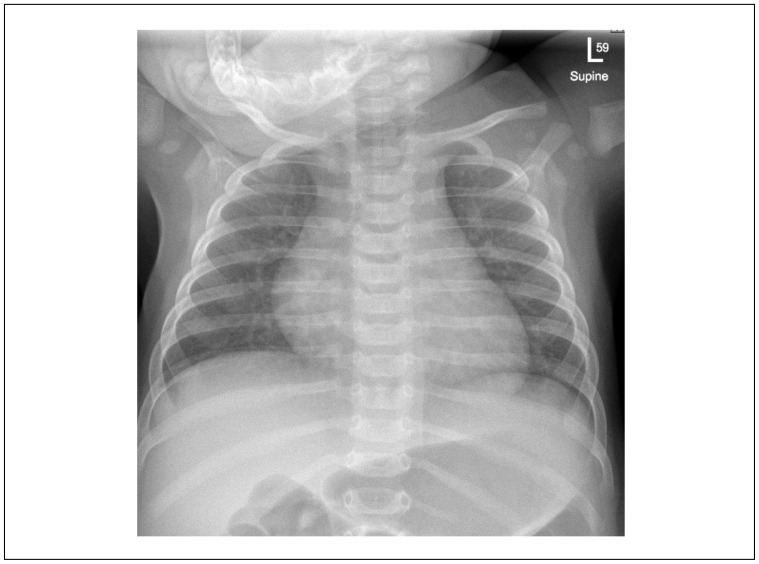
CXR^5^

## FOR EXAMINER ONLY

### Clinical Decision-Making Case: Febrile Infant

**Diagnosis:** Neonatal bacterial meningitis

**Case Summary:** The patient is a 2-week-old male born at 38 weeks, 4 days via vaginal delivery. Patient’s mother did not receive any prenatal care and was found to be positive for Group B streptococcus (GBS) at the time of birth. She was given antibiotics at the time of birth. The patient’s mother noted the fever today, measured at 100.6° F at home (axillary). The patient has been eating slightly less than usual and has had 2 wet diapers today. The patient received hepatitis B vaccination, vitamin K, and erythromycin eye ointment at the time of birth. The patient has had two pediatrician visits at 3 days old and 10 days old and has been showing appropriate weight gain. Mother denies any known sick contacts.

Patient is found to be febrile, tachycardic, and with dry mucous membranes but otherwise has a normal physical exam.

Labs are notable for an elevated white blood cell count, and lumbar puncture is consistent with bacterial meningitis (low glucose, elevated protein, elevated white blood cell count with neutrophilic predominance, and gram stain with gram positive cocci).

**Synopsis of Physical Exam:**

- Vital Signs
  ○ BP: 70/40 mmHg
  ○ HR: 190 bpm
  ○ RR: 50
  ○ T: 38.6°C
  ○ SpO2: 98% on room air
- Physical exam findings
  ○ General: Irritable but consolable infant
  ○ HEENT: Fontanelle is soft and flat, tympanic membranes with preserved light reflex and no erythema or bulging. No discharge from the eyes, mucous membranes are dry. Crying without tears.
  ○ Lungs: Increased respiratory effort. Clear breath sounds auscultated bilaterally without crackles or wheezing.
  ○ Cardiovascular: Tachycardic, regular rhythm, warm and well perfused.
  ○ Abdomen: Soft, non-distended, no hepatosplenomegaly.
  ○ Extremities: No peripheral cyanosis, no clubbing, no contractures.
  ○ Neurologic: Moving all extremities, tone present to all four extremities, Moro’s and suck reflex intact.
  ○ Skin: No rashes or jaundice.

### Clinical Decision-Making Case: Febrile Infant Examiner Script

**Case Introduction:**

“Hello Doctor, this is a clinical decision-making case. Remember, there is no role playing. You should simply respond to the questions I ask. At times, you may be asked general questions about your clinical decision-making. Additionally, at times I may interrupt you to move you through the case; this is not a reflection of your performance. You will have 15 minutes to complete the case. Do you have any questions? Alright, our time begins now”

“The patient we will be discussing is a 2-week-old male who presents to the emergency department with fever.”

Provide Learner Stimulus #1

#### HISTORY

Prompt 1:

“After you have reviewed the information, what specifically would want to find out about the history of this patient from the family?”

Scoring Guidelines:

Duration of fever, feeding difficulties, change in activity level, birth history (gestational age, vaginal or C-section, complications), maternal infections during pregnancy, recent exposures (eg, sick contacts), immunization status (Hep B vaccine received?), urinary output (diapers), prenatal care

Prompt 2:

“You indicated you would ask the patient about X. Why is this important to you?”

**ASK THIS PROMPT TWICE ABOUT TWO SEPARATE TOPICS**

Examples: Immunization status, maternal infections during pregnancy, duration of fever, urinary output, birth history, prenatal care

Scoring Guidelines:

Rationale: Learner should investigate patient immunization status, prenatal care, birth history (including maternal infections during pregnancy since this can increase risk of neonatal infection), urinary output and feeding difficulties to evaluate fluid status, and recent exposures to possible sick contacts.

“Thank you, doctor, here is the additional historical information.”

Provide Learner Stimulus #2 and read pertinent information.

#### PHYSICAL EXAMINATION

Prompt 3:

“Based on what you now know, please give me a list of specific physical examination findings you would be looking for.”

Scoring Guidelines:

Rationale: The learner should assess the fontanelle to assess for signs of dehydration or increased intracranial pressure, auscultation of the lungs to assess for pneumonia, mucous membranes to assess for signs of dehydration, cardiac murmurs to assess for underlying cardiac abnormality, abdominal tenderness to assess for intraabdominal pathology, tympanic membranes to assess for otitis media, and a skin exam to assess for rashes or jaundice.

Prompt 4:

“Doctor, why is examination of the fontanelles important during the physical exam of infants?”

Scoring Guidelines:

Rationale: The learner should assess the fontanelle to assess for signs of dehydration or increased intracranial pressure.

“Thank you, doctor, here are the physical exam findings.”

Provide Learner Stimulus #3 and read pertinent information.

#### DIFFERENTIAL DIAGNOSIS

“You are provided with the following physical exam findings:”

Prompt 5:

“Based on what you now know, what are the top three items on your differential diagnosis based on the most likely conditions?” (If more than three conditions are mentioned, say, “OK thank you. Please give me your three, and only three, most likely diagnoses.”)

Appropriate differential diagnoses include meningitis, urinary tract infection, pneumonia, viral illness, bacteremia.

Scoring Guidelines:

Rationale: Mentioning any diagnoses above, pathologies related to neonatal sepsis including meningitis, urinary tract infections, pneumonia, or other bacterial infections.

#### DIAGNOSTIC STUDIES

Prompt 6:

“Based on what you know and your working differential diagnosis, what, if any, diagnostic studies would you order?”

Prompt 7:

“Doctor, you ordered X. Why X?”

**ASK THIS PROMPT TWICE ABOUT TWO SEPARATE TOPICS**

Examples, Lumbar puncture (LP) – cell count, culture, protein, glucose, HSV (ask learner, “What studies would you order on the CSF fluid and why?”), urinalysis (UA) with urine culture, blood culture, complete blood count (CBC), basic metabolic panel (BMP), chest x-ray (CXR).

Scoring Guidelines:

Rationale: Diagnostic studies evaluating various types of bacterial infections (chest x-ray for pneumonia, urinalysis for urinary tract infection, lumbar puncture for meningitis, CBC for elevated white blood cell count, blood cultures). Learners must also voice what specifically they would like to order on the lumbar puncture (cell count, culture, protein, glucose, HSV testing).

*After the candidate responds*, Provide Learner Stimuli 4–5.

#### TREATMENT AND OTHER ACTIONS

Prompt 8:

“Based on what you now know, what treatments, if any, would you order and/or what actions, if any, would you perform?”

Scoring Guidelines:

Rationale: Antibiotics, intravenous (IV) fluids, antipyretics (acetaminophen)

Prompt 9:

“Doctor, you ordered X. Why X?”

Scoring Guidelines:

Rationale: Antibiotics should be ordered to treat the underlying infection. Intravenous fluids should be administered because patient meets sepsis criteria and is showing signs of dehydration. Antipyretics should be given because patient has a fever. Patient should ultimately be admitted to the Pediatric Intensive Care Unit (PICU).

Prompt 10:

“Doctor, in neonates that present with concerns for meningitis, what specific antibiotics should be ordered and why?

Scoring Guidelines:

Rationale: Treatment for neonatal meningitis includes IV ampicillin + gentamicin OR cefepime OR cefotaxime

#### BROAD PRINCIPLES

Prompt 11:

“Not specific to his case, doctor, but what factors do you consider in determining whether to cover the febrile neonate with acyclovir and why?”

Scoring Guidelines: Coverage for HSV (with acyclovir) should be considered when there is a maternal history of genital HSV lesions or fevers surrounding delivery and in infants who present with vesicles, seizures, or hypothermia.

#### FINAL DIAGNOSIS

Prompt 12:

“Based on everything you know about this case, what is your final diagnosis?”

Scoring Guidelines:

Meningitis

If the candidate mentions something vague such as neonatal sepsis or fever, examiner asks, “Can you be more specific about the diagnosis?”

#### DISPOSITION

Prompt 13:

“Based on what you know, what should be the disposition of this patient?”

Prompt 14:

“Why would you [admit/discharge] this patient?”

Scoring Guidelines:

Rationale: Verbalizing a disposition and level of care, and explaining it is necessary to admit the patient to the pediatric intensive care unit given the diagnosis of neonatal meningitis and need for close monitoring (necessitating a higher level of care).

#### TRANSITION OF CARE

Prompt 15:

“What specific actions would you take at the time of [admission/discharge]?

Scoring Guidelines:

Rationale: Verbalizing final actions such as explaining the diagnosis and plan to the parent and signing out the patient care to the intensive care team.

*Thank you, Doctor. That concludes this case.*

*Please tear up your notes*

## CERTIFYING EXAM ASSESSMENT

*Clinical Decision-Making Case: Febrile Infant*

Learner: _________________________________________[Table t8-jetem-11-1-ce1]

**Summative and formative comments:**

### Stimulus Inventory

**Candidate Task Sheet**

## DEBRIEFING AND EVALUATION PEARLS

### Clinical Decision-Making Case: Febrile Infant

#### Big Picture Takeaways (Why This Case Matters)

- Any fever in a neonate (<28 days) is a medical emergency until proven otherwise.^1^–^3^
- Neonates have immature immune systems and may appear deceptively well despite life-threatening infection.^1^,^3^
- The goal is early recognition, complete evaluation, prompt antibiotics, and appropriate ICU disposition.^3^

#### Core Learning Points

- Recognition
  ○ Fever ≥38.0°C (100.4°F) in a neonate (<22 days of age) automatically triggers a full sepsis evaluation.^1^–^3^
  ○ Normal exam findings (eg, flat fontanelle, consolable infant) does not exclude meningitis or bacteremia. 1,4
  ○ **Examiner Pearl:** Leaners should not anchor on appearance alone – age trumps appearance in this population.
- History That Changes Risk
  ○ Birth history: gestation age, delivery method, delivery complications.^1^,^3^
  ○ Maternal history: lack of prenatal care, Group B Strep (GBS) status, peripartum antibiotics, Herpes Simplex Virus (HSV) risk.^1^–^3^
- Absence of focal physical exam findings does *not* rule out invasive infection in neonates.^1^
- Viral illness does not eliminate the need for a full bacterial evaluation.^1^,^3^
- Diagnostic Work-up (*must include based on AAP guidelines)^1^,^3^
  ○ Blood culture*
  ○ Urinalysis with urine culture*
  ○ Lumbar puncture (LP) with cerebrospinal fluid (CSF) studies (cell count, gram stain, glucose, protein, bacterial culture)*
    ▪ Findings consistent with bacterial meningitis include elevated WBCs with neutrophilic predominance, low glucose, high protein, and positive Gram stain.^1^,^4^
  ○ HSV studies (if infant has increased HSV risk)*
  ○ +/− inflammatory markers (procalcitonin, C-reactive protein (CRP), complete blood count (can be variable early in life), chest x-ray (if neonate has any respiratory symptoms)^3^
  ○ **Examiner Pearl:** Performing or attempting the LP before antibiotics are administered when feasible is best practice, without delaying treatment in an unstable patient.^1^
- Treatment Principles
  ○ Empiric IV Antibiotics: Ampicillin + gentamicin OR ampicillin + cefotaxime^1^,^3^
  ○ Consider acyclovir if HSV risk factors present^3^
  ○ All febrile neonates require admission (recommend ICU-level care for meningitis or sepsis)^1^–^3^
